# Application of Metabolomics in the Exploration of Bioactive Compounds and Drug Development from Medicinal Fungi

**DOI:** 10.3390/metabo16080576

**Published:** 2026-08-16

**Authors:** Yan Tong, Chuyu Tang, Bing Jia, Haoxu Tang, Jinxuan Yan, Yuling Li, Xiuzhang Li

**Affiliations:** 1 State Key Laboratory of Plateau Ecology and Agriculture, Qinghai Academy of Animal and Veterinary Sciences, Qinghai University, Xining 810016, China; 2 Qinghai Provincial Grassland Engineering Technology Research Center, Xining 810016, China

**Keywords:** medicinal fungi, metabolomics, bioactive compounds, cultivation process, extraction process, pharmacological mechanisms

## Abstract

Medicinal fungi are important sources of natural medicines and are rich in a wide range of secondary metabolites with significant bioactivities, including terpenoids and alkaloids. As one of the core tools of systems biology, metabolomics has developed from simple metabolite detection into a key approach for understanding the complex life processes and medicinal value of medicinal fungi. This review summarizes recent advances in the application of metabolomics to the discovery of bioactive compounds from medicinal fungi, the optimization of cultivation and extraction processes, the analysis of pharmacological mechanisms, and the improvement of drug delivery strategies. The main methods used in metabolomics are outlined, with emphasis on the key steps of sample preparation, data acquisition, and data analysis. The review then describes how metabolomics can be used for the rapid discovery of bioactive compounds in medicinal fungi. It further explains how metabolomics can guide the precise optimization of fermentation parameters and the improvement of extraction processes by revealing the effects of environmental factors, such as light, temperature, pH, and inducers, as well as different extraction methods, on metabolic networks. In addition, this review summarizes the role of metabolomics in clarifying the molecular mechanisms by which bioactive compounds exert pharmacological effects through the modulation of specific metabolic pathways, as well as its application in optimizing dose, formulation, and route of administration. Representative studies have demonstrated that integrated metabolomic and proteomic analyses have revealed the preferential accumulation of triterpenoids, sterols, and polyphenolic compounds during the budding stage of Ganoderma lucidum. Finally, future studies should integrate metabolomics with genomics, transcriptomics, proteomics, and other omics approaches to link bioactive metabolites with their biosynthetic genes, regulatory networks, and molecular targets. In such an integrated framework, metabolomics can serve as a phenotype-oriented core for the discovery and functional characterization of therapeutic metabolites from medicinal fungi, although further experimental and clinical validation remains essential.

## 1. Introduction

### 1.1. Background

Medicinal fungi are an important reservoir of natural medicinal resources. Medicinal fungi refer mainly to macrofungi traditionally used for health promotion or investigated as sources of bioactive compounds with preventive or therapeutic potential [1]. They do not constitute a formal taxonomic group. Approximately 700 mushroom species have been reported to possess medicinal properties [2]. Taxonomically, most medicinal macrofungi belong to Basidiomycota, with a smaller proportion in Ascomycota [3], including genera such as *Ganoderma*, *Hericium*, and *Cordyceps*. Their metabolites include polysaccharides, terpenoids, alkaloids, phenolic compounds, and other bioactive molecules with anti-inflammatory, antitumor, and immunomodulatory activities [4,5,6,7,8,9,10]. The discovery, identification, and characterization of the dynamic changes in bioactive compounds in medicinal fungi are essential for advancing their practical application. However, traditional methods based on the isolation and identification of single compounds, such as bioassay-guided fractionation, can only isolate the major constituents from complex matrices, while low-abundance bioactive compounds are often overlooked, and usually only one or a few active metabolites can be obtained [11,12]. As a result, these methods are not suitable for dynamically characterizing changes in bioactive compounds. In addition, sample loss may occur at each separation step, making trace components difficult to isolate [13]. Bioactive compounds may also degrade and lose activity during the separation process [14]. By contrast, metabolomics can identify multiple components in a mixture at the same time [15,16], including unstable compounds that are easily lost during purification. It also treats all components as an integrated whole rather than a series of isolated constituents [17,18]. The metabolic network of medicinal fungi is inherently complex, and the composition and content of bioactive compounds are influenced by both the genetic background of the strain and environmental factors. Previous studies have shown that genetic differences determine the basic features of metabolic profiles [19,20], whereas variations in cultivation conditions [21,22,23,24] and growth stages [25,26] can cause marked shifts and dynamic changes in metabolite profiles. With the rapid development of modern biomedicine, the search for highly effective and low-toxicity bioactive compounds from medicinal fungi has become a major focus in drug research. Metabolomics can be used to efficiently discover new bioactive compounds from medicinal fungi, identify the optimal process parameters for high-yield production, and clarify the molecular mechanisms and optimal drug delivery strategies of these compounds, thereby promoting their translation from natural resources into clinical drugs.

This review aims to systematically summarize the applications of metabolomics in the discovery, production, and therapeutic development of bioactive compounds from medicinal fungi. Specifically, we first introduce the principal methodologies in metabolomics used for fungal bioactive metabolites. We summarize the application of metabolomics in the discovery of bioactive compounds from medicinal fungi. We then discuss the application of metabolomics in evaluating and optimizing fermentation and extraction conditions to improve the production and recovery of these compounds. Furthermore, we summarize how metabolomics contributes to elucidating their pharmacological effects and mechanisms of action. Finally, we discuss delivery strategies designed to enhance the bioavailability and therapeutic efficiency of fungal bioactive compounds.

The objects considered in this review are medicinal fungi whose bioactive metabolites have been extensively investigated using metabolomics or related analytical approaches. Particular attention is given to representative species of the genera *Ganoderma*, *Cordyceps*/*Ophiocordyceps*, and *Tremella*, as well as other medicinal fungi with sufficient metabolomic evidence concerning their bioactive constituents, cultivation responses, pharmacological activities, or therapeutic potential.

### 1.2. Literature Search and Selection Strategy

A structured literature search was conducted primarily using PubMed and the Web of Science Core Collection. No restriction was imposed on the year of publication, and all relevant records available from database inception to the final search date of 30 April 2026 were considered. The search strategy combined terms describing the research objects, including “medicinal fungi,” “medicinal fungus,” “medicinal mushrooms,” and “medicinal mushroom,” with terms related to metabolomics and fungal bioactive compounds, including “metabolomics,” “metabolomic profiling,” “metabolic profiling,” “metabolite profiling,” “bioactive compounds,” “bioactive metabolites,” and “secondary metabolites.” Names of the principal fungal genera and species discussed in this review, such as Ganoderma, Cordyceps, Ophiocordyceps, and Tremella, were also used as supplementary search terms.

To retrieve literature corresponding to the major themes of the review, these core terms were further combined with keywords related to cultivation, fermentation, metabolite production, extraction, pharmacological activities, mechanisms of action, bioavailability, and delivery systems. Boolean operators AND and OR were used to construct the search combinations. Peer-reviewed experimental studies directly relevant to the scope of the review were prioritized as the principal sources of evidence. Review articles were included to summarize established knowledge and to identify additional relevant publications through reference-list screening. Highly cited and seminal studies were also considered when they provided important conceptual, methodological, or historical contributions to the field. Citation count was used as a supplementary indicator of scientific influence rather than as an absolute inclusion threshold. Publications were excluded when they lacked a direct connection to medicinal fungi, metabolomics, bioactive compounds, or the principal themes addressed in this review. As this article is a narrative review, the final selection of literature was based on thematic relevance, scientific contribution, and the strength of the reported evidence rather than on a formal systematic-review or meta-analysis protocol.

## 2. Methodologies in Metabolomics

**Figure 1:**
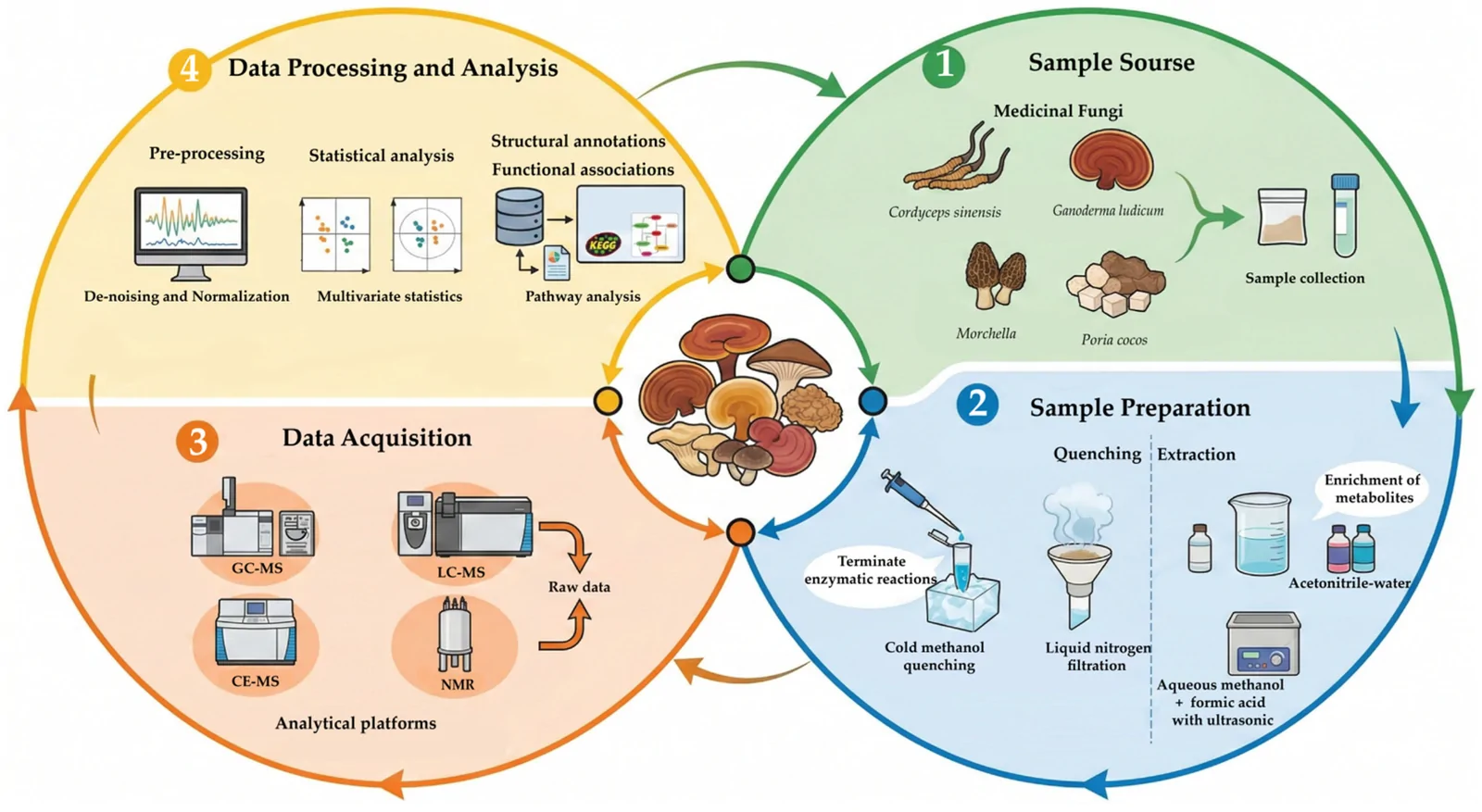
Standardized metabolomics workflow for medicinal fungi.

The paradigm of metabolomics, initially conceptualized by Nicholson in 1985, posits that physiological or pathological processes within an organism should be viewed as dynamic, systemic alterations, thereby elucidating the comprehensive landscape of metabolic activity [27]. After confirming the sample source and collecting the samples, the standardized workflow for metabolomics research encompasses critical phases including sample preparation, data acquisition, and data processing and analysis (Figure 1) [28,29].

In fungal metabolomics, common sample types mainly include mycelia and fruiting bodies [30,31]. Studies on *Sanghuangporus vaninii* have found that mycelia can serve as a substitute for fruiting bodies [32]. In medicinal fungi, samples are often harvested and immediately frozen in liquid nitrogen, stored at −80 °C, and then freeze-dried before extraction [32]. In a methodological study of the filamentous fungus *Mortierella alpina*, freeze-dried biomass provided better reproducibility and metabolite recovery than fresh biomass [33]. Air-dried herbarium mushrooms can also be used for NMR metabolomics, although drying introduces compositional shifts relative to fresh materials [34]. Sample preparation is the foundational stage of fungal metabolomics analysis. Its quality directly dictates the reliability of downstream data and the accuracy of biological interpretations, primarily involving crucial steps such as quenching and extraction [35,36]. The core objective of quenching is to rapidly terminate intracellular enzymatic reactions, thereby preserving the native metabolic composition [37]. Cold methanol quenching is the predominant method, as it efficiently extracts representative intracellular metabolites [38]. However, it risks compromising cellular integrity and inducing metabolite leakage [39,40]. Optimized protocols include utilizing a 40% aqueous methanol solution at −20 °C to enhance metabolite recovery [41], or employing the cold glycerol-saline method [42] and liquid nitrogen rapid filtration [43] to better maintain cellular robustness and facilitate the precise separation of intra- and extracellular metabolites. Furthermore, the extraction phase must achieve the high-efficiency enrichment of metabolites while preventing alterations to their physicochemical properties and chemical structures. Solvent selection is paramount, as metabolic profiles obtained via different extraction solvents exhibit significant variance, consequently impacting the metabolomics data [44]. For instance, utilizing a 60% aqueous methanol solution containing 1% formic acid coupled with ultrasonic disruption significantly increases the extraction yield of metabolites from *Scytalidium parasiticum* [45]. In contrast, the acetonitrile–water system combined with the boiling ethanol method can provide a more unbiased characterization of the metabolomic profile [43].

Data acquisition is the critical phase for capturing fungal metabolic fingerprints. Researchers must select analytical platforms based on the physicochemical attributes of the target metabolites, including gas chromatography–mass spectrometry (GC-MS), liquid chromatography–mass spectrometry (LC-MS), capillary electrophoresis–mass spectrometry (CE-MS), and nuclear magnetic resonance (NMR) spectroscopy [46,47]. These technological platforms offer distinct and complementary advantages regarding sensitivity, resolution, and structural elucidation capabilities; maximizing metabolome coverage can be achieved through multi-platform integration or orthogonal analysis [48,49,50,51]. Raw acquired data must undergo systematic pre-processing to achieve signal normalization and effectively mitigate analytical noise [52]. Subsequently, high-dimensional metabolomic datasets are analyzed using multivariate statistical approaches, including unsupervised PCA for pattern recognition and supervised PLS-DA, OPLS-DA, and sparse PLS-DA for group discrimination and feature selection; machine-learning methods such as random forest and support vector machines are also increasingly applied, with cross-validation or permutation testing used to reduce overfitting [53,54,55,56,57]. Metabolite structures are then annotated by matching accurate masses and/or MS/MS spectra against widely used resources, including HMDB, KEGG, METLIN, MassBank, and GNPS [58,59,60,61,62]. Finally, the deep metabolic regulatory network and functional associations are revealed through pathway analysis [63].

## 3. Application of Metabolomics in the Discovery of Bioactive Compounds from Medicinal Fungi

Medicinal fungi are rich in a variety of pharmacologically active substances, including terpenoids, alkaloids, nucleosides, and peptides [64]. With metabolomics techniques, researchers can rapidly discover secondary metabolites with specific bioactivities in medicinal fungi. Representative metabolites identified in medicinal fungi using metabolomic techniques are summarized in Table 1.

Terpenoids are biogenic volatile organic compounds with structures based on linked isoprene units (C5H8) [65]. According to the number of isoprene units, terpenoids can be classified into hemiterpenes, monoterpenes, diterpenes, triterpenes, and polyterpenes [66]. In studies on the bioactive components of *G. lucidum*, researchers identified two structurally novel methyl soyasaponin derivatives from liquid cultures [67]. In addition, ganoderma terpenes A–F and ganoderic acids P, D2, E2, and Q identified from *G. lucidum* showed diverse pharmacological activities. Among them, ganoderma terpene D promoted glucose uptake by activating AKT (Ser473) phosphorylation, providing a new direction for the treatment of renal fibrosis and metabolic syndrome [68]. Ganoderic acid P and ganoderic acid E2 inhibited lipopolysaccharide-induced cyclooxygenase-2 protein expression [69].

Alkaloids are a large family of natural products that includes multiple subclasses [70]. They have a wide range of activities, including antibacterial [71], antitumor [72,73], tissue repair [74], neuroprotective [75,76], anti-allergic [77], anti-inflammatory [76,78] and antioxidant effects [79,80]. Hama M. et al. isolated two new alkaloid metabolites, cordytakaoamides A and B, from *Cordyceps takaomontana* and determined their structures by NMR [81]. Cheng Y.X. et al. isolated a new pyridone alkaloid, (+)-N-deoxymilitarinone A, from *Paecilomyces farinosus* and also identified a sterol compound, 22E,4R-ergosta-7,22-diene-3 beta,5 alpha,6 beta,9 alpha-tetraol [82]. Among them, (+)-N-deoxymilitarinone A showed cytotoxicity against human neuroblastoma IMR-32 cells.

Peptide-based bioactive components in medicinal fungi are small molecules formed by amino acids linked through peptide bonds and are widely distributed in fruiting bodies, mycelia, and fermentation broths [83,84,85,86,87]. Using untargeted metabolomics combined with multivariate statistical analysis, researchers identified a unique peptide of desferriferricrocin and three new homologs from wild *Morchella* sp., and clarified their biological value as iron carriers [88]. Based on LC-MS/MS, a novel peptide (VDLPTCKGF) and its derivatives identified from *G. lucidum* were shown to have the ability to scavenge intracellular reactive oxygen species [89].

Nucleoside compounds are important bioactive components in medicinal fungi. They are diverse and include purine, adenosine, inosine, cytidine, pyrimidine, uridine, thymidine, and guanosine, among others [90]. Mishra et al. used HPTLC-MS to confirm that the water extract of *G. lucidum* and the ethanol extract of *Ophiocordyceps sinensis* were both rich in nucleoside bases [91].

Phenolic compounds constitute an important class of bioactive secondary metabolites in medicinal fungi and include phenolic acids and structurally diverse polyphenol derivatives. They have been associated with antioxidant, anti-inflammatory, antimicrobial, and antitumor activities [92]. Using LC-QTOF-MS-based metabolomics combined with molecular networking, Yao et al. identified two novel phenolic xylosides from a co-culture of *Trametes versicolor* and *Ganoderma applanatum*, demonstrating the value of metabolomics for expanding the chemical space of medicinal fungi [93]. Taken together, these findings indicate that metabolomics has accelerated the discovery and identification of bioactive components in medicinal fungi.

**Table 1:** Novel metabolites detected in medicinal fungi via metabolomic techniques.

Species	Techniques	Main Metabolites	Ref
*G. lucidum*	LC-MS/MS	soyasaponins	[67]
*G. lucidum*	^1^H NMR	ganodercins A–F	[68]
*G. lucidum*	NMR	lucidate butyl P;D-2;E-2;Q	[69]
*G. lucidum*	NMR and MS	triterpene	[94]
*C. takaomontana*	NMR	cordytakaoamides A and B	[81]
*P. farinosus*	NMR	(+)-N-deoxymilitarinone A	[82]
*Morchella* sp.	UPLC-Q-TOF-MS	peptide of desferriferricrocin	[88]
*G. lucidum*	LC-MS/MS	P1-peptide (VDLPTCKGF)	[89]
*G. lucidum; O. sinensis*	HPTLC-MS	nucleobases	[91]
*T. versicolor*; *G. applanatum*	LC-QTOF-MS	phenolic xylosides	[93]

## 4. Applications of Metabolomics in the Optimization of Cultivation Processes of Bioactive Compounds from Medicinal Fungi

The discovery of novel bioactive compounds through metabolomics is the first step toward their pharmaceutical application. To maximize the final yield of these enriched bioactive substances, cultivation processes are of great importance. The biosynthesis of secondary metabolites in medicinal fungi is highly sensitive to the fermentation environment. Metabolomics can reveal changes in metabolic pathways under disturbances in key parameters, such as carbon and nitrogen sources, temperature, pH, light, exogenous inducers, and fermentation period, thereby enabling the targeted high-yield production of desired compounds [95,96,97]. Representative metabolomic studies evaluating the effects of different cultivation conditions on the production and accumulation of bioactive metabolites in medicinal fungi are summarized in Table 2.

**Figure 2:**
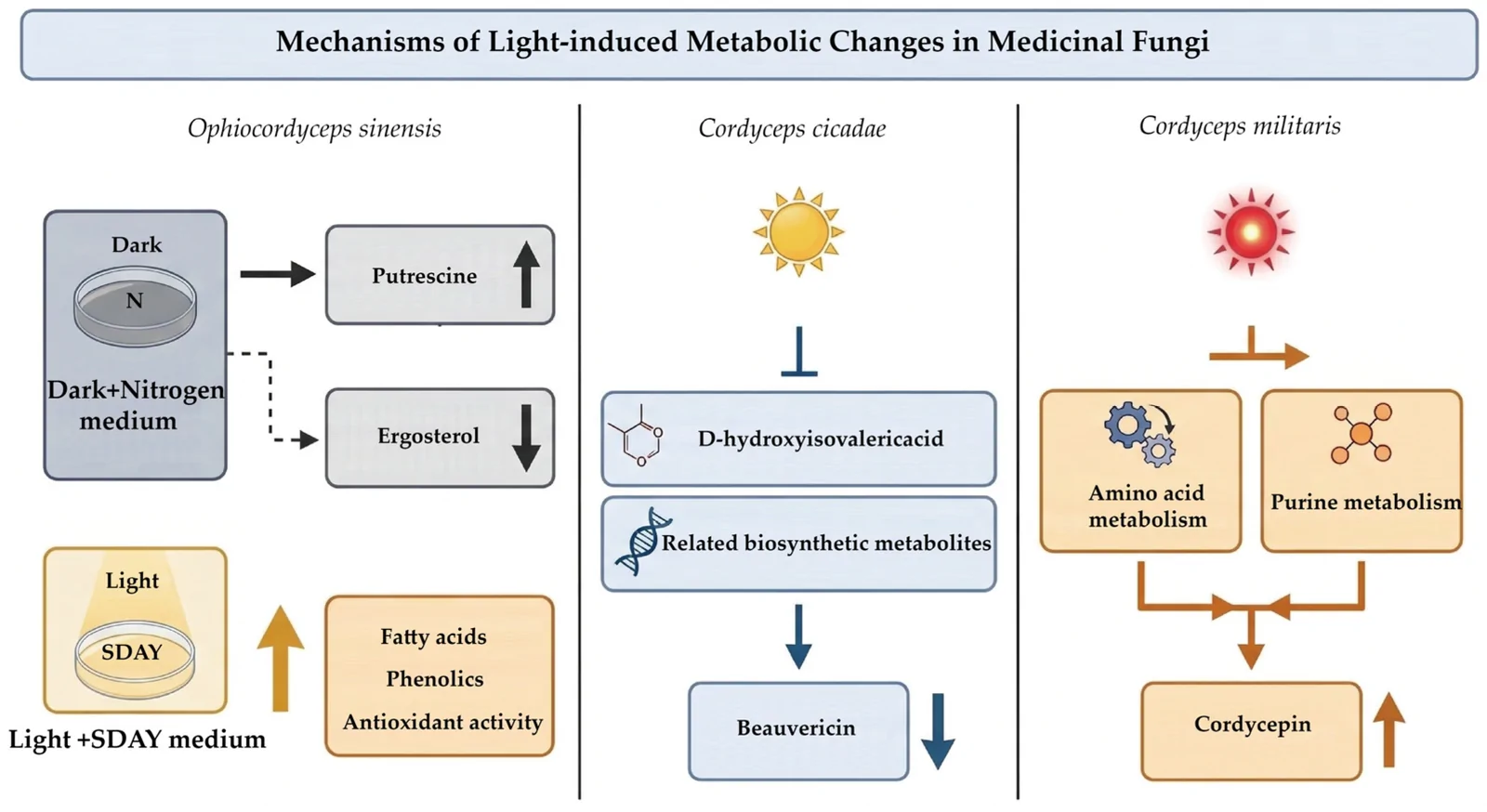
Photoregulation of Biosynthesis of Bioactive Metabolites in Medicinal Mushrooms. Among the arrows representing the increase or decrease of substance content, upward-pointing arrows indicate increased content, and downward-pointing arrows indicate decreased content.

In terms of light regulation, metabolomics studies have revealed a marked effect of light signals on metabolic networks. For example, untargeted metabolomics showed that *C. sinensis* tended to accumulate putrescine in a nitrogen-containing medium under dark conditions, which led to reduced ergosterol levels. In contrast, when grown on Sabouraud dextrose agar with yeast extract (SDAY) under light exposure (3000 lux), the contents of fatty acids and phenolic compounds, as well as antioxidant activity, reached their highest levels, indicating that light is more favorable for enhancing its value in medicinal and functional food development [96]. White light inhibited the biosynthesis of beauvericin in *Cordyceps cicadae*. Integrated transcriptomic and untargeted metabolomic analyses confirmed that this effect was associated with the downregulation of genes and metabolites involved in the biosynthesis of its precursor, D-hydroxyisovalerate, under white light conditions [95]. In addition, untargeted metabolomics showed that light stress promoted cordycepin biosynthesis in *C. militaris* by activating amino acid and purine metabolism pathways (Figure 2) [98].

With regard to physicochemical factors such as temperature and pH, secondary metabolism in the bidirectional fermentation system of *Tremella fuciformis* and *Acanthopanax trifoliatus* was significantly activated under the optimized conditions of 28 °C and pH 8, resulting in the marked accumulation of antioxidant phenolics and flavonoids [97]. Compared with the 15 °C treatment, cultivation at 25 °C significantly increased the cordycepin content in the fruiting bodies of *C. militaris*. Untargeted metabolomics analysis indicated that differential metabolites (DAMs) were mainly enriched in lipids and organic acids [99].

Changes in the types of carbon and nitrogen sources can also induce the production of unique metabolites [100]. Untargeted metabolomic results indicated that urea used as a nitrogen source could significantly promote the accumulation of biomass and extracellular ergosterol in *C. cicadae*, while reducing the intracellular ergosterol content [101].

**Table 2:** Effects of cultivation conditions on the production of bioactive metabolites.

Species	Techniques	Cultivation Parameters	Effect on Metabolite Production	Ref
*0.sinensis*	NMR and GC-MS	Light exposure	Increasing fatty acids and phenols production with light cultivation	[96]
*C. hanhua*	NMR	Light exposure	White light inhibited the biosynthesis of beauvericin	[95]
*C.militaris*	LC-MS	Light exposure	Light stress promoted cordycepin biosynthesis	[98]
*T. fuciformis*	UPLC-MS/MS	Temperature conditions; pH	Accumulation of antioxidant phenolics and flavonoids under the optimized conditions of 28 °C and pH 8	[97]
*C.militaris*	UHPLC	Temperature conditions	Increasing cordycepin production at 25 °C	[99]
*C. cicadae*	UHPLC-ESI-MS/MS	nitrogen source	Reducing the intracellular ergosterol content; increasing the extracellular ergosterol content	[101]
*G. lucidum*	LC-MS and GC-MS	exogenous inducers: MeJA	Increasing ganoderic acid content	[102]
*G. lucidum*	LC-MS	exogenous inducers: ethylene	Increasing endogenous ethylene and ganoderic acid contents	[103]
*H. sinensis*	LC-MS/MS	developmental stages	On days 24–36 of cultivation, lipids and organic acid derivatives accumulate	[104]
*C. militaris*	GC-MS	developmental stages	Accumulating cordycepin and mannitol content in the senescence process	[25]
*G. lucidum*	LC-MS	developmental stages	Increasing triterpenoids, sterols, and polyphenolic compounds content in the budding-stage	[105]

Regarding the regulation by exogenous inducers, untargeted metabolomics showed that treatment of *G. lucidum* with methyl jasmonate (MeJA) suppressed normal sugar metabolism, energy supply, and protein synthesis, while promoting the biosynthesis of secondary metabolites such as ganoderic acids [102]. Untargeted metabolomics demonstrated that exogenous ethylene can increase endogenous ethylene and ganoderic acid contents in *G. lucidum* by regulating the tricarboxylic acid cycle, polyamine metabolism, and triterpene metabolism [103].

In addition, untargeted metabolomics has characterized the chemical features of medicinal fungi at different developmental stages. In *H. sinensis*, the accumulation of metabolites such as lipids and organic acid derivatives was observed on days 24–36 of cultivation, accompanied by enhanced antioxidant capacity through increased enzymatic and non-enzymatic defense levels [104]. The senescence process of *C. militaris* is characterized by the specific enrichment of metabolites like cordycepin and mannitol, a mechanism intricately linked to the activation of the glutamine-glutamate metabolic pathway [25]. Meanwhile, the budding stage of *G. lucidum* was corroborated as the physiological zenith for the accumulation of triterpenes, sterols, and polyphenolic compounds, establishing a scientific rationale for determining the optimal harvesting window [105].

## 5. Applications of Metabolomics in the Optimization of Extraction Processes of Bioactive Compounds from Medicinal Fungi

In addition to the cultivation process, downstream extraction procedures, including raw material pretreatment, extraction techniques, and solvent selection, also determine the retention of bioactive compounds in the final product.

With regard to material pretreatment, untargeted metabolomics showed that different drying methods significantly affected the accumulation of major metabolites in *Floccularia luteovirens*, including lipids, organic acids, and their derivatives, and organic heterocyclic compounds, thereby influencing product quality [106]. Compared with oven-drying and air-drying, vacuum freeze-drying of *O. sinensis* better retains active-related metabolites such as lipids and organic oxygen compounds. These metabolic components are closely linked to antioxidant capacity [107].

In terms of extraction techniques, supercritical fluid extraction (SFE) combined with metabolite profiling not only efficiently enriches flavonoids and nucleosides from *C. sinensis*, but also produces extracts with dual effects, namely inhibition of bacterial growth through the induction of reactive oxygen species (ROS) generation and protection of cells under hypoxic conditions [108]. However, the application of metabolomics to the optimization of extraction parameters for bioactive constituents in medicinal fungi remains relatively limited.

With regard to solvent selection, GC-MS results showed that methanol extraction detected the largest number of metabolic peaks of *Phanerochaete chrysosporium* compared with biphasic extraction [109]. The ethanol extract of *G. lucidum* exhibited the highest total phenolic and flavonoid contents as well as superior antioxidant activity, acetone extracts gave the highest extraction yield, and methanol was more suitable for enriching lipophilic compounds such as lycopene [110]. Despite its widespread use in metabolomics, methanol extraction has several limitations. Methanol may react with chemically labile metabolites during extraction or storage, leading to methanolysis, esterification, or methoxylation and consequently generating artificial derivatives that are absent from the native metabolome. In particular, carboxylic acids may form methyl esters, potentially resulting in erroneous metabolite annotation and quantification [111]. Therefore, extraction conditions, solvent acidity, temperature, and storage duration should be carefully controlled to minimize methanol-derived artifacts. Overall, the optimization of extraction processes of medicinal fungi through metabolomics can markedly improve both the extraction yield and the bioactivity of active compounds.

## 6. Applications of Metabolomics in the Analysis of Pharmacological Effects of Bioactive Compounds from Medicinal Fungi

Bioactive compounds from medicinal fungi have been reported to exert multiple effects, including hypoglycemic, antidepressant, anti-inflammatory, anticancer, and gut-protective activities [112]. Metabolomics provides a systems biology perspective for elucidating the complex pharmacological actions of these compounds.

For metabolic diseases, untargeted metabolomics revealed that polysaccharides from *C. militaris* could significantly regulate amino acid metabolism, energy metabolism, and gut microbiota-related metabolism, thereby producing hypoglycemic effects [113]. In metabolic syndrome, untargeted metabolomic analysis discovered that insoluble dietary fiber from *G. lucidum* exerts hepatoprotective effects by reducing hepatic lipid deposition and the levels of biomarkers related to liver injury, while also regulating lipid metabolic disorders and improving oxidative stress [114].

In depression, the study showed that cordycepin reduced serum corticosterone levels in depressed rats and reversed chronic unpredictable mild stress (CUMS)-induced metabolic disturbances. Untargeted metabolomics further indicated that its effects were mainly associated with the alanine metabolism pathway and the tricarboxylic acid cycle [115]. Untargeted metabolomics revealed that the polysaccharide fraction CSWP-2, isolated from *C. sinensis*, mediated antidepressant effects by influencing microbiota-related metabolic processes in depressed mice and regulating metabolite levels in the ABC transporter signaling pathway [116].

In cancer, ergosterol from the mushroom *Leucocalocybe mongolica* exerted effects on breast cancer cells by regulating amino acid metabolism [117]. For gut protection, untargeted metabolomics analysis further identified indolelactic acid and 2,2-dimethylsuccinic acid as key biomarkers involved in the regulation of intestinal function in mice by polysaccharides from *G. lucidum* mycelia, and the mechanism was associated with vitamin B6 and pyrimidine metabolism [118]. By systematically analyzing the dynamic biochemical changes in organisms after drug intervention, metabolomics can clarify the pharmacological effects of these bioactive compounds.

## 7. Applications of Metabolomics in the Optimization of Drug Delivery Strategies of Bioactive Compounds from Medicinal Fungi

**Figure 3:**
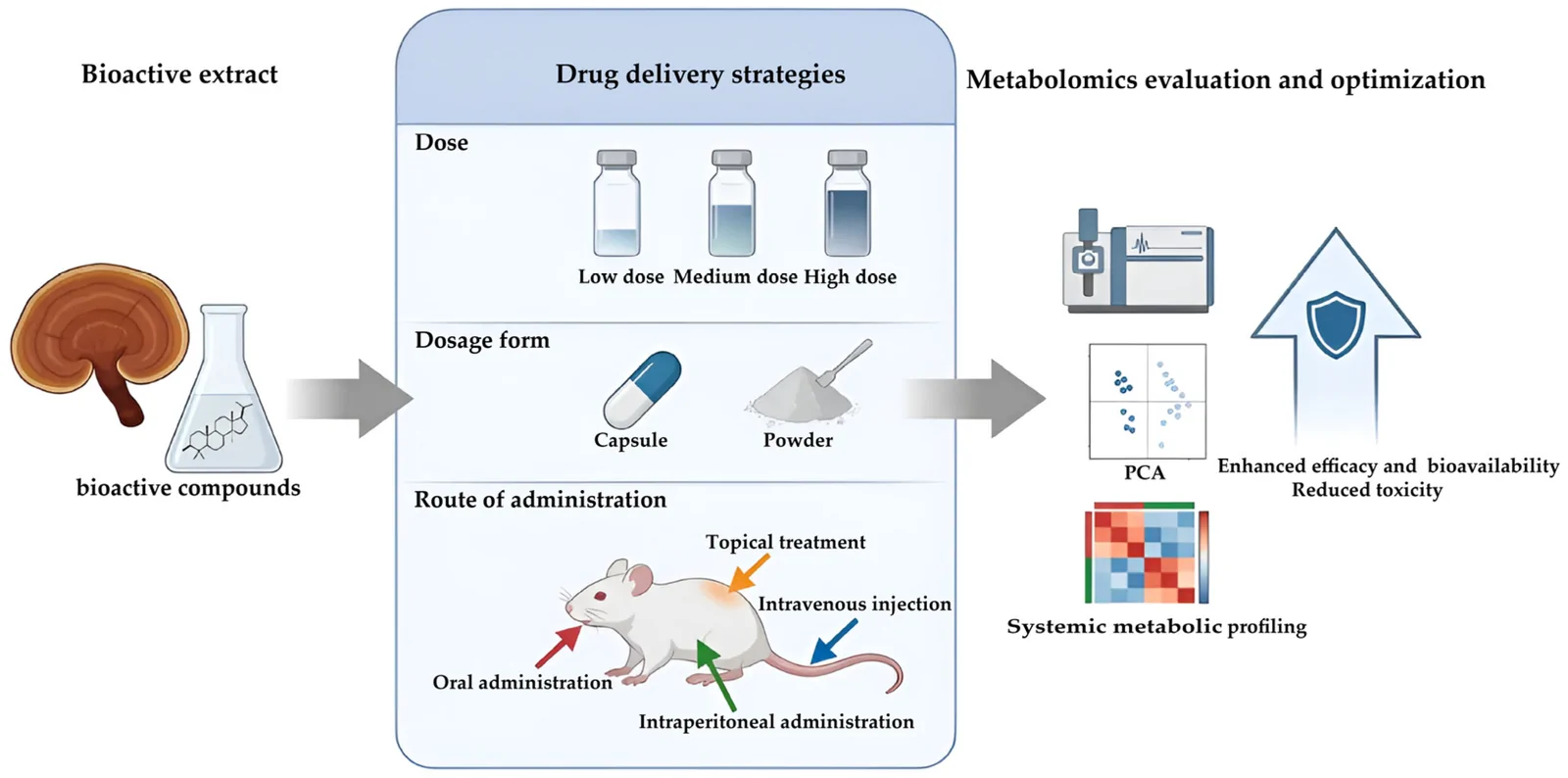
Schematic diagram of delivery strategies for bioactive components of medicinal fungi guided by metabolomics.

By dynamically monitoring metabolic responses to drug intervention, metabolomics provides key evidence for determining the optimal dose, dosage form, and route of administration of bioactive compounds, thereby improving their clinical efficacy and safety. Figure 3 shows the delivery strategies for bioactive components of medicinal fungi guided by metabolomics.

In terms of dose, metabolomics has been applied to evaluate the intervention effects of different doses of active ingredients from medicinal fungi on disease-related metabolic disorders. Both the low-dose (15 mg/kg) and high-dose (30 mg/kg) dehydrotumulosic peroxide treatment groups from *Poria cocos* improved pulmonary fibrosis in sensitized asthmatic mice. Untargeted metabolomics further revealed that these effects were closely associated with the regulation of arachidonic acid metabolism, retinol metabolism, and amino acid metabolism [119]. Meanwhile, the high-dose *Hericium erinaceus* polysaccharide group exhibited the best anti-fatigue effect in rats. Untargeted metabolomics indicated that the main metabolic pathways involved were linoleic acid metabolism, glycerol metabolism, and phenylalanine, tyrosine, and tryptophan biosynthesis [120]. These results indicate that metabolomics can help determine the optimal dosage with superior overall regulatory effects by reflecting dose-dependent metabolic remodeling characteristics.

With regard to dosage form, metabolomics can be used to evaluate the differences in the ability of different dosage forms to restore metabolic homeostasis in the body. Untargeted metabolomics identified 19 differential endogenous metabolites associated with the reversal of diabetic nephropathy in rats by the *C. sinensis* capsule [121]. It suggested that this preparation can ameliorate disease status at the overall metabolic network level. Comparative studies showed that *P. cocos* triterpenoid soft capsules significantly increased the peak plasma concentrations and area under the concentration-time curve of tumulosic acid and dehydrotumulosic acid compared with the powder form, indicating that the soft capsule dosage form could effectively enhance the maintenance of blood drug levels and improve bioavailability. However, relevant evidence is mainly derived from pharmacokinetic studies [122]. Overall, there are relatively few direct studies on optimizing dosage forms of active ingredients from medicinal fungi via metabolomics. Further systematic evaluations combining metabolomics and pharmacokinetic data are still required [123].

As far as route of administration is concerned, oral administration of *Tremella fuciformis* polysaccharides (TFPSs) was more effective than topical treatment in the treatment of dinitrofluorobenzene-induced atopic dermatitis in mice. Untargeted metabolomics revealed that it could regulate fecal metabolites and improve the composition of the intestinal flora [124]. However, in current studies on active ingredients of medicinal fungi, evidence for the optimization of administration routes mainly came from pharmacodynamics, pharmacokinetics, and safety evaluations. Zhang et al. reported that intravenous injection of ganoderenic diol F caused marked toxic reactions and rapid elimination, whereas oral and intraperitoneal administration showed good safety [125]. Compared with intravenous injection, oral administration significantly increased the blood concentration of ganoderic acid A and prolonged its half-life, which was favorable for sustained effects [126]. Gano oil, a fatty acid-rich extract from *G. lucidum*, showed stronger inhibition of paw edema at an oral dose of 50 mg/kg than topical application at 10% [127]. These results suggested that administration routes exert significant impacts on in vivo behaviors and therapeutic effects, yet their optimization lacks direct metabolomic evidence. Therefore, future studies need to integrate metabolomics, pharmacokinetics, and pharmacodynamics analyses to accurately guide the optimization of delivery strategies for active ingredients of medicinal fungi.

## 8. Discussion

Metabolomics provides greater opportunities for the discovery of structurally novel, low-abundance, yet highly bioactive compounds in medicinal fungi. Metabolomic analysis mainly relies on two analytical approaches: NMR and MS [128]. Compared with MS, NMR is capable of identifying metabolite structures, but its sensitivity is limited [129]. In metabolomic studies, MS is often coupled with GC and LC to enable more accurate metabolite identification [130]. GC-MS offers high sensitivity and reproducibility, but it usually requires derivatization of metabolites to improve their volatility and thermal stability [48]. In contrast, LC-MS does not require derivatization and can directly detect thermally unstable metabolites, making it more widely used in metabolite profiling [42,131]. Moreover, due to the high physicochemical diversity of metabolites in medicinal fungi, no single analytical platform can achieve complete coverage of the entire metabolome [132]. For example, GC-MS preferentially detects volatile or derivatizable small polar metabolites, whereas reversed-phase LC-MS is more suitable for less volatile and relatively hydrophobic compounds. Consequently, metabolites with markedly different polarity, volatility, molecular size, thermal stability, and ionization behavior are difficult to determine simultaneously under a single set of analytical conditions [133,134]. Although combining complementary platforms can substantially expand metabolome coverage, integration of data generated by different analytical methods remains challenging. GC-MS and LC-MS differ in sample preparation, chromatographic separation, ionization and detection mechanisms, retention behavior, signal response, and data structure, which complicates direct comparison and feature matching across platforms [135]. Therefore, appropriate normalization, cross-platform feature alignment, missing-value treatment, and correction for analytical and instrumental variation are required before integrated statistical interpretation [136]. In the future, with the further development of multi-platform integrated strategies, particularly the combination of high-resolution mass spectrometry, NMR, and intelligent metabolite annotation algorithms, the accuracy of structural elucidation for unknown compounds will be further improved.

However, metabolomics in medicinal fungi still has several important limitations. Reproducibility can be affected by variations in cultivation conditions, extraction procedures, instrument performance, and batch effects, making rigorous QA/QC procedures essential [137]. Data interpretation also remains challenging, because untargeted datasets often contain many redundant, partially annotated, or unknown features, and linking these signals to metabolite origins or biological pathways is frequently uncertain [63]. In addition, metabolite annotation remains a major bottleneck, as many LC-MS features cannot be confidently assigned because of incomplete spectral libraries, isomers, adducts, and in-source fragments [138]. Therefore, metabolomics results should be interpreted cautiously and supported, whenever possible, by authentic standards and standardized reporting practices. Spatial metabolomics is an emerging strategy that enables the in situ characterization of metabolite distribution with spatial resolution, thereby providing critical insight into metabolic heterogeneity within biological tissues [139]. In medicinal fungi research, this approach is expected to facilitate the investigation of bioactive constituents by revealing their spatial accumulation patterns and biosynthetic microenvironments, and thus deepen our understanding of their biosynthesis and regulation.

Different approaches, including the optimization of growth conditions such as medium composition and cultivation conditions [140,141], as well as extraction processes [142], can improve the yield and bioactivity of compounds in medicinal fungi. However, the research basis for applying metabolomics to cultivation optimization in medicinal fungi remains relatively weak, and among multi-omics approaches, metabolomics is used the least frequently [143]. Although metabolomics can identify differential metabolites, the interpretation of mechanisms and the development of actionable optimization strategies still require support from other omics approaches [103,144]. Integrating metabolomics with genomics, transcriptomics, and proteomics can link biosynthetic genes, gene expression, enzyme activity, metabolite production, and pharmacological effects. Therefore, metabolomics-guided multi-omics integration is an important future direction in medicinal fungi research. Furthermore, with the integration of artificial intelligence and machine learning approaches, the fermentation and extraction processes of medicinal fungi are expected to shift from empirical optimization toward predictive and precise regulation.

Medicinal fungi have already been applied clinically because of the diverse pharmacological effects of their secondary metabolites, particularly in oncology [145,146,147,148]. However, studies on the clinical trial level of their bioactive compounds remain limited [149]. Most evidence still comes from in vitro and animal studies, which are far from sufficient for clinical application and still fall short of standard drug development requirements [150]. Although basic research has widely confirmed the anticancer effects of *G. lucidum* [151,152], clinical evidence remains limited. A clinical study by Jin et al. found that cancer patients receiving *Ganoderma* extract in combination with anticancer therapy were 1.27 times more likely to respond to chemotherapy or radiotherapy than those not receiving *Ganoderma* extract, but the data did not demonstrate a significant effect on tumor shrinkage [153]. Therefore, in future studies, researchers should place greater emphasis on clinical metabolomics to facilitate the translation of bioactive compounds from medicinal fungi from laboratory research to clinically applicable therapies supported by evidence-based medicine.

## 9. Conclusions

By integrating analytical platforms such as LC-MS, GC-MS, CE-MS, and NMR, metabolomics provides greater opportunities for the discovery of structurally novel, low-abundance, yet highly bioactive compounds in medicinal fungi. Metabolomics can capture metabolic responses induced by environmental factors in real time, thereby providing data support for the precise optimization of fermentation parameters. At the same time, metabolomics can also be used to evaluate the effects of different extraction methods on metabolite retention, stability, and unbiased extraction capability, thus promoting the efficient extraction of bioactive compounds from medicinal fungi. By monitoring dynamic changes in endogenous metabolites in vivo, metabolomics further clarifies their molecular mechanisms of action. In addition, metabolomics can be used to assess the effects of different doses, dosage form designs, and routes of administration on pharmacological efficacy and bioavailability, thereby promoting the efficient utilization of bioactive compounds. As multi-omics and AI advance, metabolomics will improve medicinal fungi research, accelerating bioactive compound discovery and facilitating clinical translation.

## Data Availability

The supporting data for the findings of this study are available from the corresponding authors upon reasonable request.

## Abbreviations

The following abbreviations are used in this manuscript:

GC-MS	Gas chromatography–mass spectrometry
LC-MS	Liquid chromatography–mass spectrometry
CE-MS	Capillary electrophoresis–mass spectrometry
NMR	Nuclear magnetic resonance
HPTLC-MS	High-Performance Thin-Layer Chromatography–Mass Spectrometry
PCA	Principal Component Analysis
PLS-DA	Partial Least Squares Discriminant Analysis
PCX	Water-soluble polysaccharides
PCY	Water-insoluble polysaccharides
PCZ	Triterpenoid saponins
SDAY	Sabouraud dextrose agar with yeast extract
MeJA	Methyl jasmonate
SFE	Supercritical fluid extraction
ROS	Reactive oxygen species
UPLC-MS/MS	Untargeted ultra-performance liquid chromatography–high-resolution mass spectrometry
TFPSs	*Tremella fuciformis* polysaccharides
